# Mechanism of Long Noncoding RNA HOTAIR in Nucleus Pulposus Cell Autophagy and Apoptosis in Intervertebral Disc Degeneration

**DOI:** 10.1155/2022/8504601

**Published:** 2022-01-04

**Authors:** Shujun Zhang, Sheng Song, Wei Cui, Xueguang Liu, Zhenzhong Sun

**Affiliations:** ^1^Department of Spine Surgery, Wuxi 9th Affiliated Hospital of Soochow University, Suzhou, China; ^2^Department of Minimally Invasive Spine Surgery, Wuhan Pu'ai Hospital, Wuhan, China

## Abstract

**Objective:**

Intervertebral disc degeneration (IDD) contributes to cervical and lumbar diseases. Long noncoding RNAs (lncRNAs) are implicated in IDD. This study explored the mechanism of lncRNA HOTAIR in IDD.

**Methods:**

Normal and degenerative nucleus pulposus (NP) cells were isolated from NP tissues obtained in intervertebral disc surgery. Cell morphology was observed by immunocytochemistry staining and toluidine blue staining. NP cell markers were detected by RT-qPCR. Proliferation was detected by MTT assay. Autophagy-related proteins were detected by Western blot. Autophagosome was observed by monodansylcadaverine fluorescence staining. Apoptosis was detected by TUNEL staining and flow cytometry. si-HOTAIR and/or miR-148a inhibitor was introduced into degenerative NP cells. Binding relationships among HOTAIR, miR-148a, and PTEN were predicted and verified by dual-luciferase reporter assay and RNA pull-down. Finally, IDD rat models were established. Rat caudal intervertebral discs were assessed by HE staining. Expressions of HOTAIR, miR-148a, and PTEN were determined by RT-qPCR.

**Results:**

HOTAIR was highly expressed in degenerative NP cells (*p* < 0.05). si-HOTAIR inhibited degenerative NP cell apoptosis and autophagy (*p* < 0.05). HOTAIR upregulated PTEN as a sponge of miR-148a. miR-148a was poorly expressed in degenerative NP cells. miR-148a deficiency partially reversed the inhibition of si-HOTAIR on degenerative NP cell autophagy and apoptosis (all *p* < 0.05). *In vivo* assay confirmed that si-HOTAIR impeded autophagy and apoptosis in intervertebral disc tissues, thus improving pathological injury in IDD rats (all *p* < 0.05).

**Conclusion:**

LncRNA HOTAIR promoted NP cell autophagy and apoptosis via promoting PTEN expression as a ceRNA of miR-148a in IDD.

## 1. Introduction

Intervertebral discs (IVDs) are composed of nucleus pulposus (NP), annulus fibrosus (AF), and cartilage endplates, which experience a gradual degeneration under the influence of a variety of factors, such as aging and tissue damage caused by mechanical stress [1, 2]. IVD degeneration (IDD), considered a prevailing public health problem, severely affects patients' quality of life and poses huge economic burdens to families and society [3, 4]. As a highly prevalent musculoskeletal disease, IDD is associated with back and neck pain and nerve root-related pain, featured by progressive degenerative injury of intervertebral tissues and metabolic changes in other vertebral tissues [5]. The reduction of NP cell numbers, for example, in the form of apoptosis and autophagy, is tightly implicated in IDD pathogenesis [4]. Currently, the management options for IDD are primarily aimed at relieving symptoms, and therapeutic interventions involving the underlying pathology of IDD are not available [6]. Therefore, this paper sought to find novel and effective treatment approaches to improve IDD therapeutic effects.

Long noncoding RNAs (lncRNAs) are characterized by limited protein-coding ability and are involved in multiple biological processes, such as transcription, protein activity, and aging-related degenerative musculoskeletal diseases, including IDD [7]. Deregulated lncRNAs play a principal part in modulating NP cell behaviors in IDD [8]. HOX transcript antisense intergenic RNA (HOTAIR) is a kind of lncRNA associated with autophagy [9]. Meanwhile, autophagy is validated to promote IDD [10]. HOTAIR has been repeatedly proven to facilitate NP cell apoptosis and senescence by stimulating autophagy [10, 11]. Mechanically, growing studies have proposed that lncRNAs interact with microRNAs (miRNAs), thereby exerting effects on cell autophagy and apoptosis via a lncRNA-miRNA-mRNA competing endogenous RNA (ceRNA) network [12, 13]. For example, lncRNA FAM83H-AS1 can promote the growth of NP cells and maintain the tissue homeostasis of IVDs by inhibiting miR-22-3p [14]. LncRNA H19 can promote autophagy and apoptosis of NP cells, thus aggravating IDD via the miR-139/CXCR4/NF-*κ*B axis [15]. LncRNA CDKN2B-AS1 can inhibit ox-LDL-induced proliferation of vascular smooth muscle cells and promote apoptosis through the ceRNA network of CDKN2B-AS1/miR-126-5p/PTPN7 [16].

miRNAs, defined as highly conserved molecules, show deregulated expression in diverse musculoskeletal diseases, including IDD, and hold the promise as reliable biomarkers for IDD [17]. Several miRNAs have been demonstrated to inhibit NP cell autophagy and apoptosis in IDD, which can be mediated by lncRNAs [18, 19]. For instance, miR-202-5p can affect the autophagy and apoptosis of degenerative NP cells by targeting ATG7 [18]. LncRNA H19 promotes NP cell autophagy and apoptosis and aggravates IDD through the miR-139/CXCR4/NF-*κ*B axis [15]. miR-32-5p mimic contributes to NC cell proliferation and decreases apoptosis by upregulating the PI3K/AKT/mTOR pathway [20]. Additionally, a previous study has proposed that miR-148a shows intimate associations with IDD pathogenesis and close interactions with NP cells [21]. Nevertheless, little is known about the effect of miR-148a on NP cell autophagy and apoptosis in IDD.

Therefore, it is reasonable to speculate that lncRNA HOTAIR may play a role in NP cell autophagy and apoptosis with the involvement of miR-148a in IDD. Consequently, we performed a series of histological and molecular experiments to identify the underlying ceRNA network of lncRNA HOTAIR-miR-148a-mRNA in IDD, with the purpose of providing some novel therapies against AD.

## 2. Materials and Methods

### 2.1. Ethics Statement

All experimental protocols were recommended and got approval from the Ethics Committee of Wuxi 9th Affiliated Hospital of Soochow University (approval number: KT2019016). The experimental process strictly followed the approved protocol. Significant efforts were made to minimize animal numbers and suffering. Consent from patients and the Ethics Committee of our hospital was obtained prior to sample collection.

### 2.2. Isolation and Culture of NP Cells

IVDs used in this study were collected from the clinical operations in the Orthopedics Department of our hospital. IDD was assessed using T2-weighted sagittal magnetic resonance imaging (MRI) according to the modified Pfirrmann grading system before operation [22]. Based on MRI results, nondegenerative NP tissues were collected from 5 patients (3 males and 2 females; aged 12–20 years; Pfirrmann grade I) undergoing fusion surgery for adolescent idiopathic scoliosis or lumbar vertebral burst fractures, whereas severely degenerative NP tissues were collected from 5 lumbar disc herniation patients (3 males and 2 females; aged 45–58 years; Pfirrmann grades IV-V) undergoing discectomy surgery. The collected NP tissues were cut into small pieces using aseptic ophthalmic scissors and detached using phosphate-buffered saline (PBS) containing 0.025% type I collagenase (Invitrogen Inc., Carlsbad, CA, USA) for 4 h, followed by 10 min centrifugation. Next, the cell precipitates at the bottom of the centrifuge tube were dispersed and mixed. The obtained cell suspension was transferred into the culture bottle for culture. The primary NP cells were cultured in Dulbecco's modified Eagle's medium/F12 (1 : 1) medium (Gibco Company, Grand Island, NY, USA) with 10% fetal bovine serum (Gibco) in an incubator (37°C, 5% CO_2_). For about 7 days, the medium was refreshed for the first time upon complete cell adherence to the wall. When the primary cultured cells covered the monolayer (over 95% confluence), they were subcultured for phenotypic identification. An inverted phase-contrast microscope (Olympus Optical Co., Ltd., Tokyo, Japan) was used to observe cell morphology after 48 h, and NP cells of P2 generation were selected for subsequent experiments.

### 2.3. Cell Transfection

Small-interfering (si)-HOTAIR, si-negative control (NC), mimic NC, miR-148a mimic, inhibitor NC, and miR-148a inhibitor were supplied by Sangon Biotech Co., Ltd. (Shanghai, China). All transfections were conducted following Lipofectamine 2000 (Invitrogen) instructions. Cells were cultured in an incubator for subsequent experiments.

### 2.4. Immunocytochemistry (ICC) Staining

After cleaning, acid soaking, and sterilizing, the cover slides were placed in the cell culture dish and added with cell suspension for 24–48 h conventional culture. After the cover slides were covered by cells, the culture medium was removed. Then cells were rinsed with PBS, fixed in 4% formaldehyde for 15 min, and incubated with 0.3% Triton-100 for 20 min. Following PBS washing, the cells underwent 30 min incubation with 1% bovine serum albumin (BSA) and then 1 h incubation with primary antibody Collagen II (1 : 500, ab34712, Abcam Inc., Cambridge, MA, USA) at 37°C. Following PBS washing, the cells were reacted with secondary antibody immunoglobulin G (IgG) (1 : 2000, ab205718, Abcam) for 30 min in a wet box, followed by PBS washing. Next, the cells were conventionally stained with 2,4-diaminobutyric acid and counterstained with hematoxylin. Following dehydration and clearance, the cells were sealed and observed under a microscope (TS100, Nikon Instruments, Chiyoda-ku, Tokyo, Japan).

### 2.5. Toluidine Blue Staining

NP cells underwent 2 min PBS washing and 30 min fixing with 4% paraformaldehyde room temperature, followed by PBS washing. Then the cells were immersed and stained with 1% toluidine blue (Sigma-Aldrich, Merck KGaA, Darmstadt, Germany) for 2 h and counterstained with hematoxylin. Next, the excess staining solution was removed using 95% ethanol. Finally, the cells were cleared in xylene, sealed using neutral gum, and observed and photographed under a microscope.

### 2.6. 3-(4,5-Dimethylthiazol-2-yl)-2,5-diphenyltetrazolium Bromide (MTT) Assay

Cells in the exponential phase of growth were harvested and added with 5 g/L MTT solution (20 *μ*L, Gibco) at 24/48/72/96 h of culture, respectively. After 4 h further culture and the removal of the supernatant, each well was added with 150 *μ*L dimethyl sulfoxide, followed by shaking for full dissolution of crystals. Next, the optical density (OD) value at 490 nm of each well was determined using a microplate reader (Rayto Life Science Co., Ltd., Shenzhen, Guangdong, China), with 5 duplicated wells for each group. The experiment was repeatedly performed three times.

### 2.7. Reverse Transcription Quantitative Polymerase Chain Reaction (RT-qPCR)

Total RNA was extracted using TRIzol (Invitrogen) one-step method, and high-quality RNA was verified using UV spectrophotometer and formaldehyde denaturation electrophoresis. The cDNA was synthesized through reverse transcription from l *μ*g RNA using AMV reverse transcriptase. SYBR Green Mix (Thermo Fisher Scientific Inc., Waltham, MA, USA) was used for qPCR. PCR primer design and synthesis were accomplished by Sangon (Table 1). The reaction system included cDNA (1.0 *μ*L), 2 × SYBR Green Mix (10 *μ*L), forward primer (10 *μ*M; 0.5 *μ*L), and reverse primer (10 *μ*M; 0.5 *μ*L), and RNase free water was used to supplement to 20 *μ*L. Reaction conditions were 94°C for 5 min (predenaturation) and then 40 cycles of 94°C for 40 s (denaturation), 60°C for 40 s (annealing), and 72°C for 1 min (extending), followed by 10 min extending at 72°C. U6 served as an internal parameter for miR-148a, and glyceraldehyde-3-phosphate dehydrogenase (GAPDH) served as an internal parameter for HOTAIR, aggrecan, Collagen II, and PTEN. The 2^−ΔΔCt^ method was used for data analysis.

### 2.8. Western Blot (WB) Analysis

Cell protein was extracted, and protein concentration was determined as per the instructions of the bicinchoninic acid kit (Boster Biological Technology Co., Ltd., Wuhan, Hubei, China). Following the addition of loading buffer (30 *μ*g/well), the extracted protein underwent 10 min boiling at 95°C. The protein was then subjected to 10% polyacrylamide gel (Boster) electrophoresis (voltage changed from 80 V to 120 V) for separation and then loaded onto polyvinylidene fluoride membranes via wet transfer at a voltage of 100 mV for 45–70 min. Following 1 h blocking with 5% BSA at room temperature, the membranes underwent overnight incubation at 4°C with primary antibodies phosphatase and tensin homolog (PTEN) (1 : 1000, ab267787, Abcam), light chain 3 (LC3)I, LC3II (ZSGB-Bio Co., Ltd, Beijing, China), p62 (2 *μ*g/mL, ab56416, Abcam), Beclin-1 (1 : 2000, ab207612, Abcam), and GADPH (1 : 2500, ab9485, Abcam). Following 3 washes with Tris-buffered saline-Tween 20 (5 min per wash), the membranes underwent 1 h incubation with a secondary antibody IgG (1 : 2000, ab205718, Abcam) at room temperature. The membranes were washed (3 times, 5 min each) and developed using chemiluminescence reagent and GelDOC EZ Imager (Bio-Rad Inc., Hercules, CA, USA). ImageJ software (National Institutes of Health, Bethesda, Maryland, USA) was utilized to analyze the gray value of the target bands.

### 2.9. Monodansylcadaverine (MDC) Fluorescence Staining

NP cells in each group underwent 4 h culture in an incubator (37°C, 5% CO_2_). After medium removal, the cells were rinsed twice with PBS and added with MDC staining solution (0.1 mM, Sigma-Aldrich) for 45–60 min incubation at 37°C. After the removal of the MDC staining solution, the cells were subjected to 15 min fixing at room temperature in 4% paraformaldehyde and 2 washes with PBS. Next, cells were observed and photographed using a fluorescence microscope (excitation wavelength = 425 nm; emission wavelength = 525 nm) after drying. A total of 100 cells were randomly selected in the field (200 ×), and the number of cells containing punctate fluorescent autophagic vesicles was counted (the NP cells with 5 or more punctate fluorescent autophagic vesicles were counted once). The autophagy percentage = cells containing autophagic vesicles/100 cells × 100%. The final value was the average of three repeated tests.

### 2.10. Terminal Deoxynucleotidyl Transferase- (TdT-) Mediated dUTP Nick End Labeling (TUNEL) Staining

The cells (1 × 10^5^/well) were seeded into 6-well plates and cultured for 24 h. After stable attachment to the slide, the cells underwent PBS washing and 30 min fixing with 4% paraformaldehyde, followed by 30 min incubation with 0.1% Triton X-100 solution. Based on the instructions of a TUNEL kit, the enzyme reaction solution (tube 1) and the labeling solution (tube 2) (1 : 9) were added to the cover slides, with the NC group only added with the labeling solution (tube 2) as control. Next, the cells underwent 60 min incubation away from light at 37°C under saturated humidity. Hoechst working solution was added to stain the nucleus for 20 min incubation at room temperature. Finally, cover slides were sealed using an antifade mounting medium, and a fluorescence microscope (Olympus) was utilized for cell apoptosis observation.

### 2.11. Flow Cytometry

The cells were detached using trypsin and made into a single-cell suspension. After centrifugation (4°C, 100 g, 5 min) and the supernatant removal, the cells were washed twice with precooled PBS, suspended in the binding buffer, and added with 5 *μ*L Annexin V and 1 *μ*L propidium iodide working solution for 15 min reaction at room temperature avoiding light. Next, 300 *μ*L binding buffer was added and gently mixed. Approximately 10000 cells were determined utilizing a flow cytometer, and then the percentage of apoptotic cells was calculated.

### 2.12. RNA-Fluorescence In Situ Hybridization (FISH)

The subcellular localization of lncRNA HOTAIR in NP cells was determined with firm compliance to the instructions of the RNAscope Multiplex Assay kit (Thermo Fisher). The HOTAIR fluorescent probe was designed and provided by the kit's manufacturer [23].

### 2.13. Nuclear and Cytoplasmic Fractionation Assay

The nuclear extract was prepared based on the manufacturer's instructions using the NE-PER nuclear and cytoplasmic extraction kit (Thermo Fisher). Cell precipitates were subjected to vortex oscillation for 15 s and suspended in 1 mL cytosol extraction reagent (CER)I (10 times the volume of cell precipitation) supplemented with phenylmethylsulfonyl fluoride (PMSF). Next, the suspension was incubated on ice for 10 min and then added with CERII (CERI: CERII: nuclear extraction reagent (NER) = 200 : 11 : 100) for 5 s vortex oscillation, followed by 1 min incubation on ice and centrifugation (16000 g, 5 min). The supernatant (cytoplasmic extract) was transferred to the precooled tube. The insoluble precipitates containing coarse nuclei underwent 15 s vortex oscillation and resuspension in 1 mL NER added with PMSF (PMSF: NER = 1 : 100), followed by 40 min incubation on ice. Vortex was carried out for 15 s every 10 min. After centrifugation (16000 g, 10 min), the obtained supernatant was nuclear extract. HOTAIR expression in nuclear and cytoplasmic extracts was detected using RT-qPCR.

### 2.14. Dual-Luciferase Reporter Gene Assay

Bioinformatics software and websites were utilized to predict the binding sites of HOTAIR with miR-148a and miR-148a with PTEN. HOTAIR 3′UTR and PTEN 3′UTR sequences containing miR-148a binding site were synthesized, and HOTAIR-wild type (WT) and PTEN-WT plasmids were constructed. Based on these plasmids, the binding sites were mutated, and HOTAIR-mutant (MUT) and PTEN-MUT were constructed. After that, HOTAIR-WT, HOTAIR-MUT, PTEN-WT, and PTEN-MUT plasmids were mixed and cotransfected with miR-NC and miR-148a mimic plasmids, respectively, into 293T cells (American Type Culture Collection, Manassas, Virginia, USA). After 48 h transfection, the cells were collected and lysed. The luciferase activity was determined using a luciferase detection kit (BioVision, San Francisco, CA, USA) and GloMax 20/20 luminometer (Promega, Madison, WI, USA).

### 2.15. RNA Pull-Down Assay

An Eppendorf (EP) tube without RNase was added with 50 *μ*L magnetic beads, 20 mM Tris solution (pH = 7.5), 1 × RNA capture buffer, and 50 pmol biotin-labeled miR-143, respectively, for 2 h incubation at 37°C. Afterwards, the incubated EP tube was added with 2 *μ*L DNase I for 15 min incubation at 37°C. The reaction was terminated by the addition of 2 *μ*L ethylenediaminetetraacetic acid (EDTA) (0.2 M, pH = 8.0). Biotin-labeled RNA (1 *μ*g) was added with a proper amount of structure buffer (10 mM Tris, pH = 7.0; 0.1 M KCl; 10 mM MgCl_2_), heated at 95°C for 2 min, put on the ice bath for 3 min, and rested at room temperature for 30 min. Afterwards, the magnetic bead-RNA mixture was added with cell lysis buffer (containing about 1 mg protein). An appropriate amount of RNase inhibitor was added to the lysis buffer, which was then placed at room temperature for 1 h and centrifuged at low speed with the supernatant recovered as the NC of the system, followed by 3 washes with Wash Buffer II (1 mL per wash). The liquid was added or dripped along the wall, turned up and down slowly, and mixed thoroughly. Blowing using pipetting was not allowed. Finally, the RNA product was obtained, and HOTAIR expression was detected using RT-qPCR.

### 2.16. Animal Treatment

A total of 32 adult male Sprague-Dawley rats (230–260 g, 7–10 weeks) (Shanghai Branch of Beijing Vital River Laboratory Animal Technology Co., Ltd. (Beijing, China); SYXK (Shanghai) 2017–0014)) were kept in the standard animal room at 18–22°C and provided with freely available food and water under a light/dark cycle. All animals were euthanized by intraperitoneal injection of pentobarbital sodium (≥100 mg/kg).

### 2.17. Establishment of IDD Animal Model

A total of 32 rats were randomly allocated to the sham group, IDD + PBS group, IDD + si-NC group, and IDD + si-HOTAIR group, with 8 rats in each group. All the animals were anesthetized using 40 mg/kg pentobarbital sodium (2% (w/v)). The IVDs (Co6-7, Co7-8, and Co8-9) in the tailbone of the rats that needed IDD modeling were located by palpation and then confirmed using radiography. The AF (Co6-7 and Co8-9, about 4 mm deep) was punctured using a needle (27 G) through the caudal skin, parallel to the endplates. Next, all needles were rotated 360° axially and placed for 1 min [10]. Subsequently, 2 *μ*L PBS, si-NC, or si-HOTAIR was slowly injected into the target tailbone, and the needle was kept for 2 min. All animals were allowed free loading and movement. All rats were euthanized 4 weeks after IDD surgery. Caudal IVDs and adjacent vertebral bodies were collected from 4 rats for histological evaluation. The IVD tissues of the remaining 4 rats were homogenized for subsequent WB and RT-qPCR experimentations.

### 2.18. Histological Evaluation

Four weeks after IDD operation, the rats were euthanized, and the IVDs and adjacent vertebral bodies were resected and fixed with 10% formalin. The samples were decalcified in 10% EDTA for 30 days, embedded in paraffin, and then sectioned (5 *μ*m) using a microtome (Leica RM 2145, Leica Microsystems, Nussloch, Germany). Hematoxylin and eosin (HE) and TUNEL staining were used for histological evaluation. The histological evaluation was conducted employing previously published methods [24].

### 2.19. Statistical Analysis

Data were analyzed utilizing SPSS 21.0 (IBM Corp. Armonk, NY, USA). Kolmogorov–Smirnov test verified that data were in the normal distribution. The results were represented as mean ± standard deviation. The independent sample *t*-test was utilized for comparative analysis between two groups, and one-way analysis of variance (ANOVA) was utilized for comparative analysis among multiple groups followed by Tukey's multiple comparisons test. *p* < 0.05 indicated statistically significant difference; *p* < 0.01 represented highly statistically significant difference.

## 3. Results

### 3.1. HOTAIR Was Highly Expressed in NP Cells of IDD Patients

**Figure 1:**
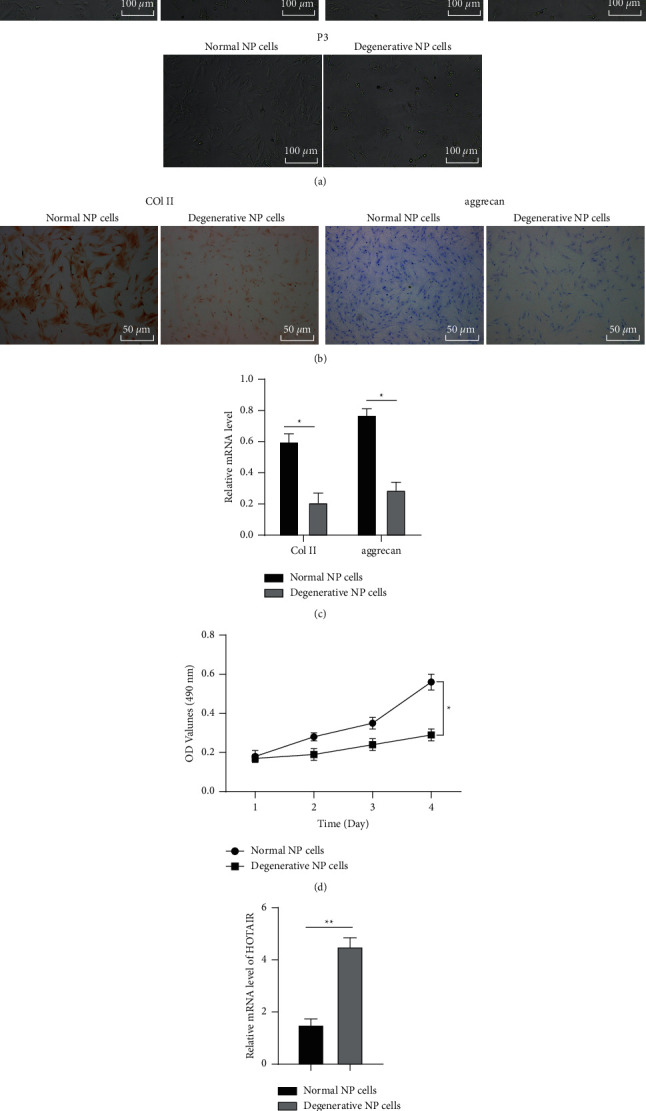
HOTAIR is highly expressed in NP cells of IDD patients. (a) The morphological changes of normal and degenerative NP cells were observed using an inverted phase-contrast microscope. (b) Normal and degenerative NP cells were observed by ICC and toluidine blue staining. (c) Collagen II and aggrecan mRNA expressions in normal and degenerative NP cells were detected using RT-qPCR. (d) MTT assay was used to detect the proliferation of normal and degenerative NP cells of P2 generation. (e) RT-qPCR was used to detect the expression of HOTAIR in normal and degenerative NP cells. The experiment was repeated three times, and the data were expressed as mean ± standard deviation. Data comparisons between two groups were analyzed using independent *t*-test. ^*∗*^*p* < 0.05; ^*∗∗*^*p* < 0.01.

LncRNAs are critical in IDD pathogenesis [11]. To study the role of lncRNA HOTAIR in NP cells, we first isolated and cultured normal and degenerative NP cells from NP tissue samples obtained from clinical disc surgery. NP cells of P1, P2, and P3 generations were compared. NP cells of P2 generation with full morphology, abundant cytoplasm, strong refraction and metabolism, and the strongest proliferation ability were selected for subsequent experiments (Figure 1(a)). ICC staining of Collagen II showed that the cytoplasm of normal NP cells was stained with typical brown yellow, and as it approached the nucleus, the staining became darker, while the cytoplasmic staining of degenerative NP cells was lighter than that of normal NP cells, showing light yellow (Figure 1(b)). Similar results were also found in toluidine blue staining, which showed blue color in normal NP cell cytoplasm and light blue color in degenerative NP cell cytoplasm (Figure 1(b)), together with reduced aggrecan generation compared with normal NP cells. To further confirm the results of ICC and toluidine blue staining, we detected Collagen II and aggrecan mRNA expression in normal and degenerative NP cells of P2 generation using RT-qPCR. Collagen II and aggrecan mRNA expressions were remarkably reduced in degenerative NP cells relative to those in normal NP cells (both *p* < 0.05) (Figure 1(c)). Normal and degenerative NP cell proliferation was determined using MTT assay, which demonstrated increased OD value of the two groups with the extension of culture time and lower OD values in degenerative NP cells than those in normal NP cells on days 2–4 (*p* < 0.05) (Figure 1(d)), indicating the lower proliferation ability than normal NP cells. The above results were consistent with the growth characteristics of degenerative NP cells. Finally, HOTAIR expression in normal and degenerative NP cells was detected by RT-qPCR, which found remarkably elevated HOTAIR expression in degenerative NP cells (*p* < 0.01) (Figure 1(e)).

### 3.2. HOTAIR Knockdown Inhibited Autophagy and Apoptosis of Degenerative NP Cells

**Figure 2:**
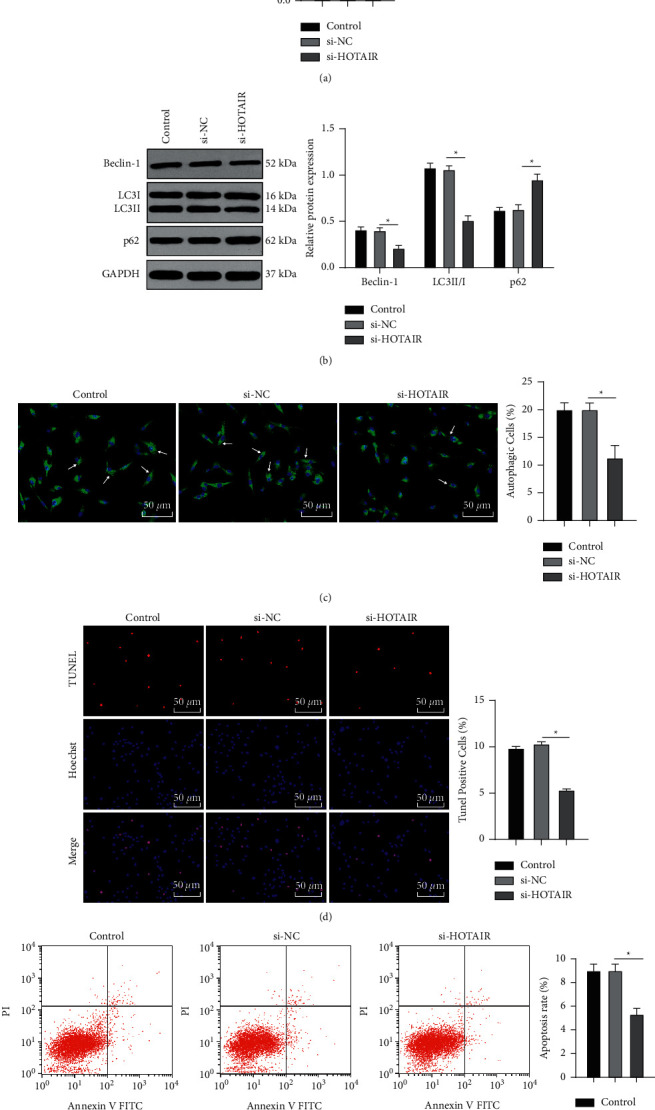
HOTAIR knockdown inhibits autophagy and apoptosis of degenerative NP cells. si-HOTAIR was transfected into degenerative NP cells. (a) The expression of HOTAIR was detected using RT-qPCR. (b) Levels of autophagy-related proteins (LC3II/I, p62, and Beclin-1) were detected using WB. (c) The number of autophagic cells was detected using MDC staining. (d, e) The apoptosis of degenerative NP cells in each group was detected using TUNEL staining and flow cytometry. The experiment was repeated three times, and the data were expressed as mean ± standard deviation. Data comparisons among multiple groups were analyzed using one-way ANOVA, followed by Tukey's multiple comparisons test. ^*∗*^*p* < 0.05.

To study the specific role of HOTAIR in autophagy and apoptosis of NP cells, we reduced HOTAIR expression by transfecting si-HOTAIR into degenerative NP cells, and the successful transfection was verified using RT-qPCR (*p* < 0.05) (Figure 2(a)). Levels of autophagy-related proteins (LC3II/I, p62, and Beclin-1) were then detected using WB. si-HOTAIR treatment reduced LC3II/I and Beclin-1 levels and elevated p62 levels (all *p* < 0.05) (Figure 2(b)). According to MDC fluorescence staining results, degenerative NP cells transfected with si-HOTAIR exhibited markedly decreased autophagic cells (*p* < 0.05) (Figure 2(c)), suggesting inhibition of degenerative NP cell autophagy by HOTAIR knockdown.

As shown by TUNEL staining and flow cytometry results, si-HOTAIR-treated degenerative NP cells showed reduced apoptosis rates (all *p* < 0.05) (Figures 2(d) and 2(e)). These results indicated that silencing HOTAIR impeded autophagy and apoptosis of degenerative NP cells.

### 3.3. HOTAIR Acting as a ceRNA Competitively Bound to miR-148a to Upregulate PTEN Expression

**Figure 3:**
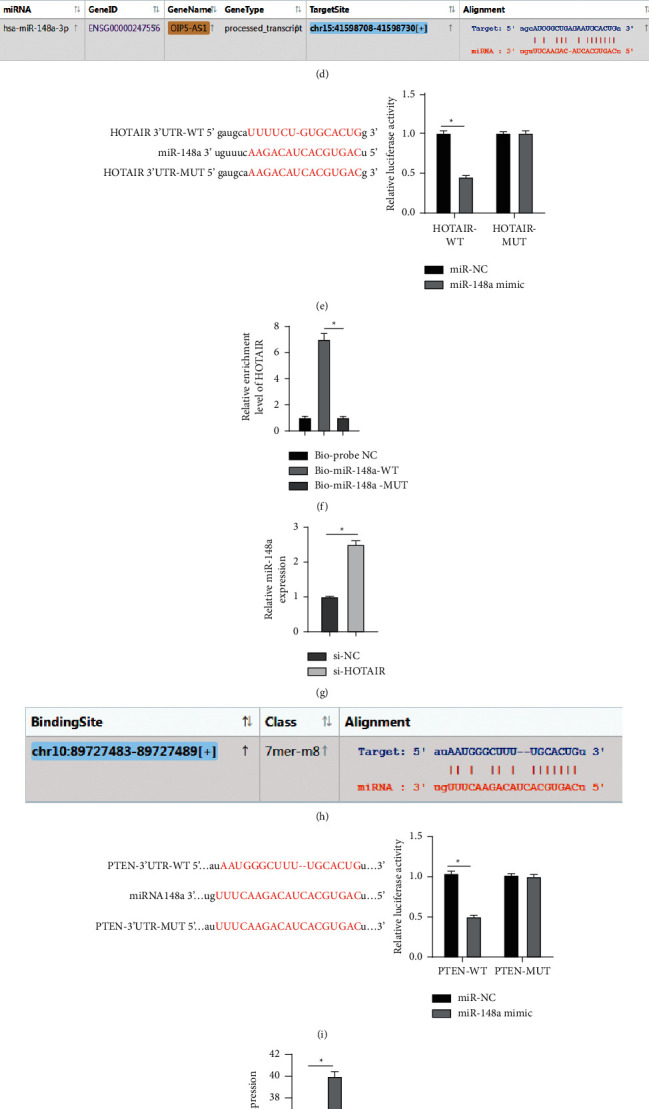
HOTAIR acting as a ceRNA competitively binds to miR-148a to upregulate PTEN mRNA expression. (a) The lncRNA HOTAIR location was predicted through the database (https://www.csbio.sjtu.edu.cn/bioinf/lncLocator/). (b) The fluorescence localization of HOTAIR in NP cells was detected using RNA-FISH. (c) The expression of HOTAIR was detected using RT-qPCR after nuclear and cytoplasmic fractionation assay. (d) The binding sites of HOTAIR and miR-148a were predicted through the bioinformatics website (https://starbase.sysu.edu.cn/agoClipRNA.php?source=lncRNA). (e) Dual-luciferase reporter gene assay was used to verify the binding relationship between HOTAIR and miR-148a. (f) RNA pull-down was used to verify the binding relationship between HOTAIR and miR-148a in NP cells. (g) RT-qPCR was used to detect miR-148a expression after HOTAIR knockdown. (h) Bioinformatics website (http://starbase.sysu.edu.cn/agoClipRNA.php?source=lncRNA) was used to predict the binding sites of miR-148a and PTEN. (i) Dual-luciferase reporter gene assay was used to verify the binding relationship between miR-148a and PTEN. (j) RT-qPCR was used to detect the expression of miR-148a. (k) RT-qPCR was used to detect PTEN mRNA expression. The experiment was repeated three times, and the data were expressed as mean ± standard deviation. Data in panels E/G/I were analyzed using *t*-test; data in panels C/F/J/K were analyzed using one-way ANOVA, followed by Tukey's multiple comparisons test. ^*∗*^*p* < 0.05; ^*∗∗*^*p* < 0.01.

The action mechanism of lncRNA depends on its location in cells. To explore the mechanism of HOTAIR in autophagy and apoptosis of NP cells, we first predicted lncRNA HOTAIR location through the database (https://www.csbio.sjtu.edu.cn/bioinf/lncLocator/) [25]. HOTAIR was mainly located in the cytoplasm (Figure 3(a)). Next, HOTAIR subcellular localization in NP cells was analyzed using RNA-FISH and nuclear and cytoplasmic fractionation assays, which verified that HOTAIR was mainly located in NP cell cytoplasm (both *p* < 0.01) (Figures 3(b) and 3(c)). The above findings suggested that HOTAIR may regulate NP cells through the mechanism of ceRNA.

Subsequently, we predicted that many miRNAs can bind to HOTAIR through the database (https://starbase.sysu.edu.cn/agoClipRNA.php?source=lncRNA) [26], among which miR-148a is common in IDD tissues and related to the IDD mechanism [27]. Therefore, we speculated that miR-148a may be a key miR in the ceRNA network of HOTAIR. Next, based on the binding sites of HOTAIR and miR-148a (Figure 3(d)), the binding relationship between the two was verified using dual-luciferase assay and RNA pull-down (all *p* < 0.05) (Figures 3(e) and 3(f)). Finally, miR-148a expression in degenerative NP cells transfected with si-HOTAIR was determined using RT-qPCR, which showed a negative correlation with HOTAIR expression (*p* < 0.05) (Figure 3(g)).

Starbase database (https://starbase.sysu.edu.cn/agoClipRNA.php?source=lncRNA) predicted that miR-148a has targeting relationships with multiple genes, among which PTEN is associated with IDD [28]. Therefore, according to the binding sites of miR-148a and PTEN 3′UTR (Figure 3(h)), we verified that miR-148a targeted PTEN using a dual-luciferase assay (*p* < 0.05) (Figure 3(i)). In addition, miR-148a mimic or miR-148a inhibitor was transfected into degenerative NP cells, which markedly increased or decreased miR-148a expression, correspondingly (both *p* < 0.05) (Figure 3(j)), along with notably reduced or elevated mRNA expression of PTEN. Moreover, PTEN mRNA expression was dramatically reduced after si-HOTAIR treatment (all *p* < 0.05) (Figure 3(k)). From all above, HOTAIR functioning as a ceRNA competitively bound to miR-148a and upregulated PTEN mRNA expression.

### 3.4. miR-148a Knockdown Reversed the Inhibition of si-HOTAIR on Degenerative NP Cell Autophagy and Apoptosis

**Figure 4:**
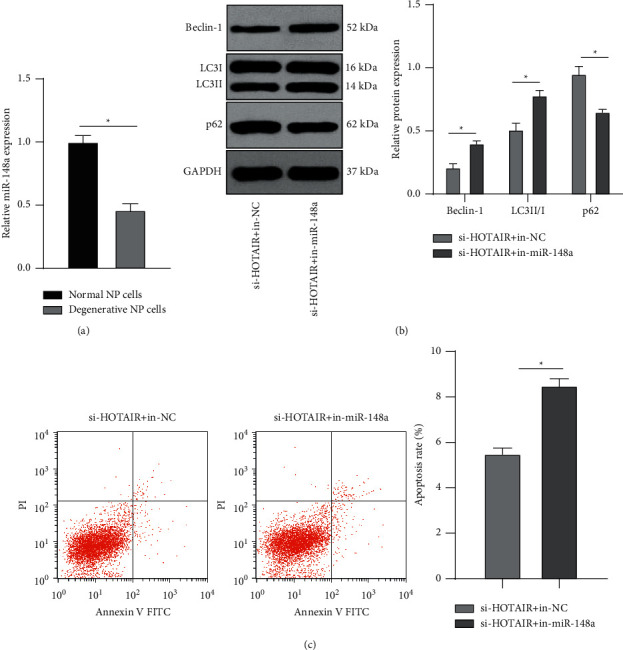
miR-148a knockdown reverses the inhibition of si-HOTAIR on the autophagy and apoptosis of degenerative NP cells. miR-148a inhibitor/inhibitor NC was transfected into degenerative NP cells with si-HOTAIR treatment group (si-HOTAIR + in-NC group/si-HOTAIR + in-miR-148a group). (a) The expression of miR-148a in normal and degenerative NP cells was detected using RT-qPCR. (b) Levels of autophagy-related proteins (p62, LC3II/I, and Beclin-1) were detected using WB. (c) Apoptosis was detected using flow cytometry. The cell experiment was repeated three times, and the data were expressed as mean ± standard deviation. Data comparisons between two groups were analyzed using *t*-test. ^*∗*^*p* < 0.05.

Firstly, miR-148a expression in normal and degenerative NP cells was measured. According to RT-qPCR results, miR-148a expression in degenerative NP cells was remarkably reduced relative to that in normal NP cells (*p* < 0.05) (Figure 4(a)). Next, combined experiments were conducted to confirm the above ceRNA network. miR-148a inhibitor/inhibitor NC was transfected into si-HOTAIR-treated degenerative NP cells (si-HOTAIR + in-NC group/si-HOTAIR + in-miR-148a group). Levels of autophagy-related proteins (p62, LC3II/I, and Beclin-1) were detected using WB, which showed diminished p62 levels, and augmented LC3II/I and Beclin-1 levels after si-HOTAIR + in-miR-148a treatment, indicating that miR-148a knockdown reversed the effect of si-HOTAIR on autophagy of degenerative NP cells (all *p* < 0.05) (Figure 4(b)). According to flow cytometry results, degenerative NP cells exhibited noticeably elevated apoptosis rate after si-HOTAIR + in-miR-148a treatment (*p* < 0.05) (Figure 4(c)). Collectively, silencing miR-148a reversed the impact of HOTAIR on degenerative NP cell autophagy and apoptosis.

### 3.5. Silencing HOTAIR Inhibited Autophagy and Apoptosis and Improved Pathological Damage of NP Cells in IDD Rats

**Figure 5:**
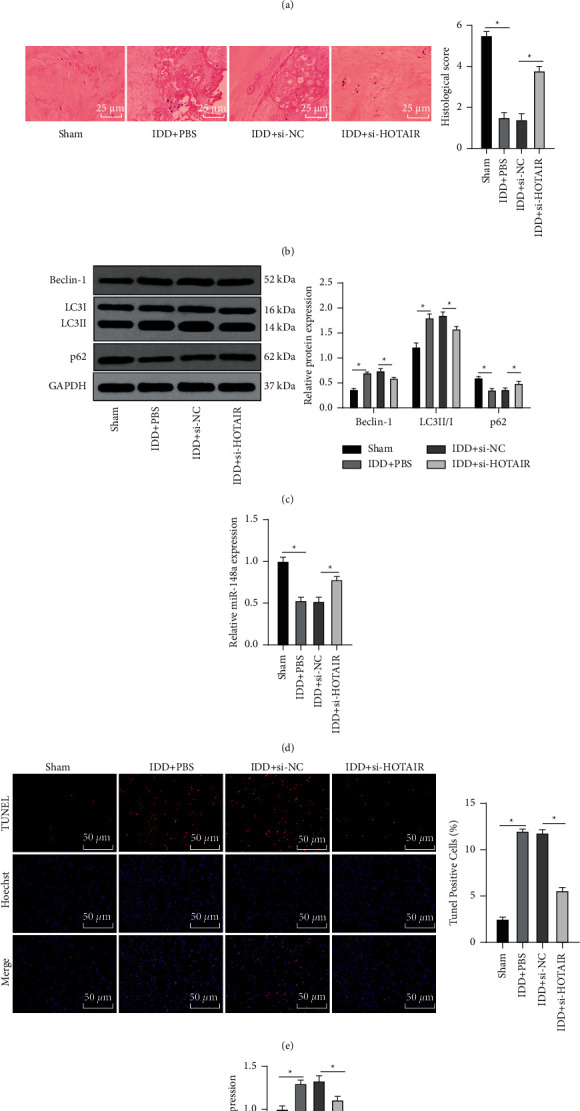
Silencing HOTAIR inhibits autophagy and apoptosis and improves pathological damage of NP cells in IDD rats. (a) RT-qPCR was used to detect the expression of HOTAIR in IVD tissues. (b) The pathological condition of NP tissues in each group was evaluated using HE staining and scored by histological evaluation. (c) WB was used to detect the levels of autophagy-related proteins (p62, LC3II/I, and Beclin-1) in IVD tissues. (d). RT-qPCR was used to detect the expression of miR-148a in IVD tissues. (e) TUNEL staining was used to detect apoptosis. (f) RT-qPCR was used to detect PTEN mRNA expression in IVD tissues. The experiment was repeated three times, and the data were expressed as mean ± standard deviation. Data comparisons among multigroups were analyzed using one-way ANOVA followed by Tukey's multiple comparisons test. ^*∗*^*p* < 0.05.

Our *in vitro* experiments confirmed that HOTAIR knockdown inhibited degenerative NP cell autophagy and apoptosis, which was further verified *in vivo*. IDD rats were firstly induced, and we found that HOTAIR expression in NP cells of IDD rats was notably increased and then successfully reduced after si-HOTAIR treatment (all *p* < 0.05) (Figure 5(a)). The pathological condition of NP tissues in each group was evaluated using HE staining and scored by histological evaluation. IDD rats exhibited remarkable NP degeneration and notably reduced histological score compared with sham-operated rats, while attenuated NP degeneration and an increased histological score were observed after si-HOTAIR treatment (*p* < 0.05) (Figure 5(b)). The above results indicated that silencing HOTAIR improved pathological damage of IDD rats.

Next, levels of autophagy-related proteins (p62, LC3II/I, and Beclin-1) in rat IVD tissues were detected. As shown by WB results, si-HOTAIR-treated IDD rats exhibited notably reduced LC3II/I and Beclin-1 levels and increased p62 levels, which indicated the attenuation of autophagy in IDD rats by si-HOTAIR (all *p* < 0.05) (Figure 5(c)). TUNEL staining revealed that the apoptosis rate of IDD rats was reduced after si-HOTAIR treatment (*p* < 0.05) (Figure 5(e)). Furthermore, as indicated by RT-qPCR results, compared with the IDD + si-NC group, the IDD + si-HOTAIR group showed markedly elevated miR-148a expression (*p* < 0.05) (Figure 5(d)) and notably reduced PTEN mRNA expression (*p* < 0.05) (Figure 5(f)). Overall, the above *in vitro* and *in vivo* experiment results revealed that HOTAIR knockdown elevated miR-148a expression to downregulate PTEN expression, thereby inhibiting degenerative NP cell autophagy and apoptosis and improving IDD.

## 4. Discussion

IDD contributes largely to low back pain and even disability [6]. LncRNAs are intrinsically related to NP cell functions in IDD [8]. This study demonstrated that lncRNA HOTAIR functioned as a ceRNA of miR-148a to promote PTEN mRNA expression, thereby inducing NP cell autophagy and apoptosis in IDD.

LncRNAs are implicated in IDD pathogenesis [7]. Deregulated NP cells participate in IDD initiation and progression and can be regulated by lncRNAs in IDD [8]. A previous study has pointed out that lncRNA HOTAIR plays a regulatory role in NP cells in IDD [11]. According to our results, NP cells in IDD patients exhibited degenerative characteristics, and HOTAIR expression was remarkably elevated in degenerative NP cells. Consistently, HOTAIR expression in NP cells shows a positive correlation with IDD grade [10]. Thereafter, we silenced HOTAIR expression in degenerative NP cells to further verify the effects of HOTAIR in IDD. It is well established that the imbalance between autophagy and apoptosis of NP cells makes great contributions to IDD progression [2, 29]. As presented by our findings, si-HOTAIR treatment reduced LC3II/I and Beclin-1 levels and elevated p62 levels in degenerative NP cells, accompanied by dramatically reduced apoptosis rate. In previous studies, increased LC3II/I and Beclin-1 levels and reduced p62 levels in NP cells are strong indicators of autophagy [30, 31]. In agreement with these, HOTAIR knockdown attenuates the degeneration process and IDD symptoms and inhibits NP cell apoptosis [11]. Our *in vivo* experiments further verified that silencing HOTAIR inhibited degenerative NP cell autophagy and apoptosis in IDD rats, with manifestations of noticeably attenuated NP cell autophagy and apoptosis and markedly improved pathological damage in IDD rats. Likewise, in a prior study, HOTAIR has been proven to stimulate autophagy and promote apoptosis of NP cells, thereby aggravating IDD [10]. Overall, we highlighted that silencing HOTAIR impeded autophagy and apoptosis of degenerative NP cells.

As has been pointed out previously, lncRNAs play a nonneglectable role in IDD via a lncRNA-miRNA-mRNA ceRNA network [13]. To explore the regulatory downstream mechanism of lncRNA HOTAIR, we predicted and identified miR-148a as a key miR in the ceRNA network of lncRNA HOTAIR in degenerative NP cells. Consistently, miR-148a shows a close relation with IDD progression [21]. Numerous studies have demonstrated that HOTAIR acts as a ceRNA of miR-148a in multiple diseases [32, 33]. Furthermore, we validated PTEN as a target gene for miR-148a in degenerative NP cells, and PTEN mRNA expression showed a positive correlation with HOTAIR expression and a negative correlation with miR-148a expression, which were further proven in IDD rats *in vivo*. Likewise, a previous study has proposed that PTEN is tightly involved in IDD development [34]. PTEN is overexpressed in degenerative NP cells and can induce NP cell apoptosis [28]. Accumulating studies documented the targeting relationship between miR-148a and PTEN in diverse disorders [35, 36]. Additionally, HOTAIR can drive osteoarthritis progression, which is associated with the regulation of PTEN [25]. Briefly, we proved that HOTAIR acting as a ceRNA competitively bound to miR-148a to upregulate PTEN mRNA expression in degenerative NP cells.

Subsequently, we investigated the role of miR-148a in degenerative NP cells. We observed that miR-148a expression in degenerative NP cells was remarkably reduced, and miR-148a knockdown reversed the inhibitory effect of si-HOTAIR on the autophagy and apoptosis of degenerative NP cells. Likewise, in a prior study, miR-148a showed an aberrant downregulation in IDD [27]. miR-148-3p also protects against cell apoptosis in osteoarthritis [37]. Collectively, silencing miR-148a reversed the inhibition of si-HOTAIR on degenerative NP cell autophagy and apoptosis.

All in all, this study supported that lncRNA HOTAIR knockdown suppressed NP cell autophagy and apoptosis via the miR-148a/PTEN axis in IDD. These results discovered a novel lncRNA-based therapy for IDD patients. However, HOTAIR was chosen as the research object after literature consulting, which is not the best strategy to screen key factors. It might be of more representative significance to screen other potential regulatory lncRNAs or HOTAIR-mediated miRNAs in IDD development using the bioinformatics method (such as high throughput screening). This study simply revealed the role of the HOTAIR-regulated ceRNA network in NP cell autophagy and apoptosis in IDD, yet the clinical application of the HOTAIR/miR-148a/PTEN axis in IDD needs to be further verified.

## Figures and Tables

**Table 1:** Primer sequences for RT-qPCR.

Gene	Primer
HOTAIR	F: 5′-CATTCTGCCCTGATTTCCG-3′
	R: 5′-ATCCGTTCCATTCCACTGCG-3′

miR-148a	F: 5′-ATGCTCAGTGCACTACAGAA-3′
	R: 5′-GTGCAGGGTCCGAGGT-3′

Aggrecan	F: 5′-TGAGCGGCAGCACTTTGAC-3′
	R: 5′-TGAGTACAGGAGGCTTGAGG-3′

Collagen II	F: 5′-TCCAGATGACCTTCCTACGC-3′
	R: 5′-TCCAGATGACCTTCCTACGC-3′

PTEN	F: 5′-CCATAACCCACCACAG-3′
	R: 5′-CAGTCCGTCCTTTC-3′

GAPDH	F: 5′-GGGAGCCAAAAGGGTCAT-3′
	R: 5′-GAGTCCTTCCACGATACCAA-3′

U6	F: 5′-CGCTTCGGCAGCACATATAC-3′
	R: 5′-AATATGGAACGCTTCACGA-3′

## Data Availability

The data generated or analyzed during this study are included in this published article.
